# Benefits of a digital health technology for older nursing home residents. A de-novo cost-effectiveness model for digital health technologies to aid in the assessment of toileting and containment care needs

**DOI:** 10.1371/journal.pone.0295846

**Published:** 2024-01-02

**Authors:** Katharina Abraham, Tim Andre Kanters, Adrian Stuart Wagg, Nicole Huige, Edward Hutt, Maiwenn Johanna Al

**Affiliations:** 1 Institute for Medical Technology Assessment, Erasmus University Rotterdam, Rotterdam, The Netherlands; 2 Erasmus School of Health Policy & Management, Erasmus University Rotterdam, Rotterdam, The Netherlands; 3 Department of Medicine, University of Alberta, Edmonton, Alberta, Canada; 4 Gothenburg Continence Research Centre, Sahlgrenska Academy, University of Gothenburg, Gothenburg, Sweden; 5 Essity Hygiene and Health AB, Gothenburg, Sweden; 6 Medica Market Access Ltd, Tonbridge, United Kingdom; University of Verona, ITALY

## Abstract

The aim of this study was first, to introduce a comprehensive, *de-novo* health economic (HE) model incorporating the full range of activities involved in toileting and containment care (T&CC) for people with incontinence, capturing all the potential benefits and costs of existing and future Digital Health Technologies (DHT) aimed at improving continence care, for both residential care and home care. Second, to use this novel model to evaluate the cost-effectiveness of the DHT TENA SmartCare Identifi in the implementation of person-centred continence care (PCCC), compared with conventional continence care for Canadian nursing home residents. The *de-novo* HE model was designed to evaluate technologies across different care settings from the perspective of several stakeholders. Health states were based on six care need profiles with increasing need for toileting assistance, three care stages with varying degrees of toileting success, and five levels of skin health. The main outcomes were incremental costs and quality-adjusted life years. The effectiveness of the TENA SmartCare Identifi was based primarily on trial data combined with literature and expert opinion where necessary. Costs were reported in CAD 2020. After 2 years, 21% of residents in the DHT group received mainly toileting as their continence care strategy compared with 12% in the conventional care group. Conversely, with the DHT 15% of residents rely mainly on absorbent products for incontinence care, compared with 40% with conventional care. On average, residents lived for 2.34 years, during which the DHT resulted in a small gain in quality-adjusted life years of 0.015 and overall cost-savings of $1,467 per resident compared with conventional care. Most cost-savings were achieved through reduced costs for absorbent products. Since most, if not all, stakeholders gain from use of the DHT-assisted PCCC, widespread use in Canadian residential care facilities should be considered, and similar assessments for other countries encouraged.

## Introduction

Urinary incontinence (UI) places a high burden on the individual, their caregivers, and society and is expected to have increasing impact as the population ages [1–3]. Individuals with incontinence experience a range of physical and psychological effects. There is a high prevalence of UI in nursing homes, with studies indicating between 43% and 77% of residents with this condition [4]. Skin conditions such as incontinence-associated dermatitis (IAD) (perineal dermatitis) are present in up to 50% of residents, resulting in inflammation and tissue damage to the vulva, perineum, perineal region, and buttocks as well as increasing the likelihood of developing pressure ulcers (PU) [5–7]. IAD results in lower quality of life (QoL) due to pain and discomfort. Regardless of its effect on skin health, incontinence adversely affects QoL resulting in decreased life satisfaction scores, depression, and poorer self-rated health, increasing with increasing severity of UI. This decrease in QoL is observed in nursing home residents at all levels of cognitive impairment [8]. UI is also associated with substantial economic burden, mostly related to medical visits, treatments and absorbent products [9]. Care for PU as a result of IAD is estimated to cost up to €470 per patient per day [6].

Continence care which aims to manage symptoms of differing severity can be broadly divided into either toileting or containment strategies [10,11]. Where possible, toileting is promoted as the primary strategy for managing incontinence, combined with state-of-the-art perineal hygiene [12]. Absorbent products are used as an additional protection for incontinence episodes between toilet visits and for those who are unable to successfully toilet consistently due to physical or cognitive impairment (thus ensuring a situation of *contained incontinence*) [13]. Toileting strategies include set toileting times and toileting programs, such as prompted voiding [10]. Bladder diaries, manually kept by the caregiver, record the resident’s voiding and involuntary leakage patterns to inform toileting times. In nursing homes, staff and financial resources to provide toileting assistance are often lacking [14]. Consequently, the focus is often on containment care only. Various types of containment product exist but absorbent products are most commonly used [15,16]. There is a wide range of product types, with the most widely used being pads, pull up pants and (belted) briefs. There are also different sizes and absorbency levels to meet different care needs. However, in practice a narrower range is often used due to a lack of understanding as to which product is best suited to an individual’s needs, or as a result of purchasing practices restricting availability [17]. Assessment of residents need for containment care is often based on ‘after the fact’ data such as leakage incidents. Anecdotal statements from nursing home staff show that high absorbency products designed for protection at night are often used during the day to avoid leakage and consequent labour-intensive change of bedlinen, clothes, and cleaning of the resident. Nursing home staff consider toileting and containment care (T&CC) assessments and the provision of T&CC to be difficult and time-consuming [18]. The challenging nature of these assessments, combined with the lack of staff and financial resources, mean that the wishes and individual needs of the residents often play only a marginal role in the management of their UI [14,19].

Improvements in T&CC have historically been achieved through implementing a person-centred continence care (PCCC) approach that improves quality of care and QoL for residents [20]. With PCCC, individuals’ values and preferences are elicited and shape all aspects of their care [21]. In recent years Digital Health Technologies (DHTs) have been developed to help tailor T&CC to the individual’s needs by improving the accuracy of continence assessments to support personalised care delivery, leading to greater toileting success, thereby reducing skin problems and improving QoL [19,22,23]. Reductions in acquisition costs for absorbent products and their associated disposal as well as reductions in the time spent on continence management could also be achieved [22,24,25].

While evidence for the effectiveness of DHT is building there is a lack of economic data on their implementation. Three cost-effectiveness studies [26–28] were found on PCCC interventions, which focus mainly on cure to increase QoL of individuals. However, in persons for whom cure is not achieved, person-centred T&CC strategies present an opportunity to improve QoL. Also, no economic study could be found on both toileting and containment strategies with support of digital health technologies to improve QoL. In addition, two of the three cost-effectiveness studies were based on empirical data regarding costs and effects.

Therefore, the aim of this study was twofold. First, to introduce a comprehensive, *de-novo* health economic model incorporating the full range of activities involved in T&CC and capturing all the potential benefits and costs of existing and future medical device technologies aimed at improving continence care, for both residential care (long-term care provided in a care home) and home care (professional or informal care provided at home). Second, to use this novel innovative model to evaluate the cost-effectiveness of the DHT TENA SmartCare Identifi to support PCCC compared with current continence care, in a residential care setting.

## Health economic model for person-centred toileting and containment care

### Model population

The population of interest for our model are older adults (>65 years) with urinary or faecal incontinence, which comprises a heterogenous population [29]. Within this population some manage their symptoms independently whilst others require assistance. Of those who require assistance some cannot express their toileting needs due to cognitive impairment, such as Alzheimer’s disease or related dementias (ADRD), or they might be unable to fulfil their toileting needs due to limited mobility or dexterity. Others are limited in both cognitive and physical ability. Given this heterogeneity, six need profiles were developed by T&CC experts based on two main characteristics: 1) cognitive ability to express toileting needs and 2) physical ability and dexterity to toilet. This resulted in six need profiles. Group 1 (G1) consists of the care independent population and Group 2 to Group 6 (G2-G6) represent the care dependent population with different levels of T&CC assistance (see Table 1).

**Table 1:** Definition population Group 1 to Group 6.

G1	Those with incontinence who do not need care from others, although they may benefit from support in choosing appropriate absorbent products.
G2	Those with either moderately reduced mobility or occasional mild confusion, able to dress themselves, and requiring occasional support but mostly achieving full toileting. Absorbent products provide reassurance in case toileting is not successful.
G3	Those with a combination of possible occasional confusion and either significantly reduced mobility or the inability to dress themselves, requiring assistance on many or most toileting occasions. A greater dependence on caregiver assistance, which may not always be available when needed, means the individual relies more often on containment than those in Group 2.
G4	Those with a significant degree of confusion, meaning a greater need for carer involvement to identify when to toilet as well as to support with the toileting itself, with more reliance on containment than those in Group 3. Mobility may be reduced to the degree that the assistance of a maximum of one caregiver is required. The individual may or may not need assistance with dressing. Successful toileting is still achievable with a high degree of personalised care.
G5	Those for whom restricted mobility is the driver of T&CC care requirements. The need for an active mechanical lift in addition to support from a single caregiver to transfer to the toilet requires more caregiver time and results in a greater reliance on containment than those in Group 4. In addition, the individual may have any degree of confusion. Reduced mobility makes the individual likely to require support with dressing. Successful toileting is still achievable with caregiver support and a high degree of personalised care.
G6	This population is defined by further reduction in mobility beyond Group 5. Toileting may still be possible with use of a passive mechanical lift and two caregivers to allow use of a bedside commode, but advanced immobility means that most urine voids require containment. The individual is likely to need help with dressing. This degree of immobility may be accompanied by any degree of confusion.

In the model, it is possible to define both for residential care and for home care the proportions of each need profile. It is to be expected that in most countries, no patients from group 1 will be in residential care. Likewise, for many countries it is plausible that only a small percentage of the home care population consists of patients in group 5 or 6.

### Current continence care

Current continence care in nursing homes and home care differs across countries and care providers. To make sure that our model would be able to incorporate the current care, we conducted individual interviews with several leading experts in the field of UI and related care strategies (N = 7; from Canada (N = 3), Germany (N = 1), and the Netherlands (N = 3); two having an MD and are geriatric specialists, four having a background in nursing, of whom two now working and teaching in Nursing Science, and one being an expert in occupational therapy). They described several common T&CC strategies employed in nursing homes including toileting times set by the care facility, residents calling for assistance using a nearby bell, prompting (based on a bladder diary kept by staff), and routine wet checks. Some care providers base the choice of containment product on individual needs of the resident whilst others make use of a standard product depending on the level of person-centredness, knowledge of the professional caregivers, and possibly health insurance coverage. Thus, current T&CC strategies range from largely person-centred care (Level 1) to largely conventional care (Level 3), with some countries and care providers providing a mix of person-centred and conventional care (Level 2). To provide insight on what aspects would drive person-centredness and how the level of person-centredness could be assessed, a questionnaire to determine the level of person-centeredness in nursing homes is provided in S1 File. For care independent individuals in the home setting, T&CC strategies are determined by individual preference, provision of information by continence specialists, and, potentially, health insurance coverage.

### Health and economic outcomes

The health economic model provides various intermediate outcomes considered most relevant to the different stakeholders on the T&CC pathway and with greatest potential impact on QoL [30]. According to the interviewed experts, the most important outcome is the level of toileting event success, defined as voids that land in the toilet rather than in the absorbent product [23]. Since prevalent and new or worsening UI has been found to decrease QoL in the frail, functionally and cognitively impaired older adult [8], it may be reasonable to expect that improved toileting event success might improve QoL in care recipients. In addition, skin health may improve when more patient-centred care is given, also leading to a QoL increase. By improving toileting success, it might also be possible to reduce some of the adverse consequences of UI, such as pressure ulcers, urinary tract infections (UTI), constipation, and fractures from falls, potentially improving QoL. Finally, the environmental impact may be deemed relevant if an intervention has the potential to reduce the number of absorbent products needed.

Note that the model was implemented into Excel such that other intermediate outcomes can be added that may be relevant to a specific setting, country or population.

Finally, the main aim of this study was to develop a health economic model that may be used by regional or national reimbursement agencies, as well as payers. Thus, the primary outcomes of the model are presented in terms of total costs, life years (LYs), quality-adjusted life years (QALYs) and the incremental cost-effectiveness ratio (ICER) [31–33]. QALYs are calculated based on LYs and health-related QoL. This QoL is expressed as a utility score, where a 0 utility reflects being dead and 1 perfect health. These utility scores are calculated with the EQ-5D-5L questionnaire [34]. This is a generic (i.e. not disease specific) questionnaire with five dimensions, each with five levels (no problems to severe problems). The EQ-5D has previously been validated in UI [35]. In addition, in the model QoL can be estimated using the Alzheimer’s disease-related quality of life scale (QoL-AD), a specific 13-item questionnaire designed to provide both a self-report and a caregiver-report of the QoL for individuals who have been diagnosed with Alzheimer’s Disease (AD) [36]. The costs included in the economic evaluation encompass the costs for the new DHT intervention being assessed, all health care related costs (such as those related to carer time, absorbent products, treatment of adverse consequences of UI, and laundry), and costs of waste disposal. In addition, it is possible to take costs into account that are borne by the care recipient (out-of-pocket costs)

However, depending on the setting for which an economic evaluation is carried out, non-medical costs such as those for informal care or productivity losses can also be incorporated.

### Analytical approach

**Fig 1:**
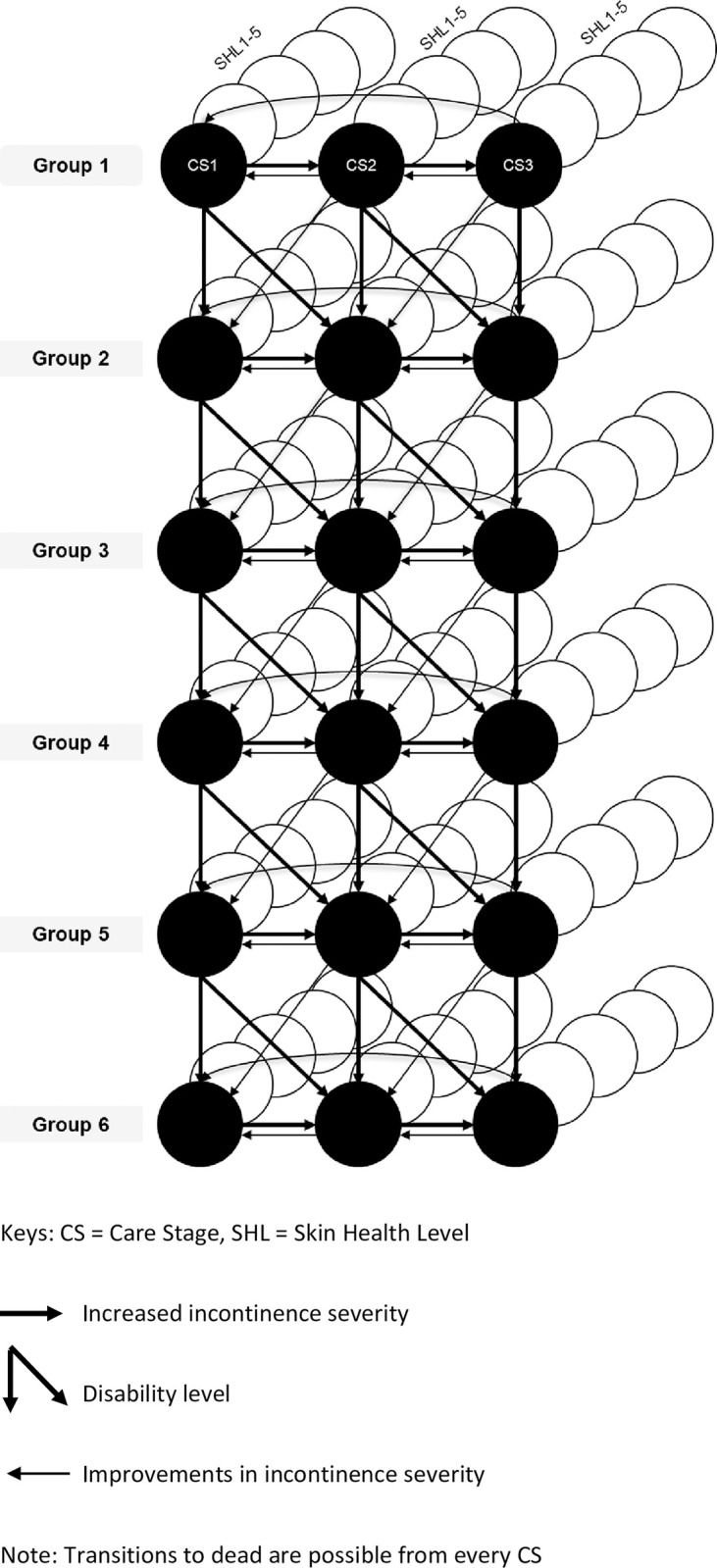
Decision model structure.

To capture all relevant costs and benefits associated with PCCC for the T&CC pathway a *de-novo* Markov decision analytical model was developed in Excel (Fig 1). The potential improvement in QoL by tailoring continence care is conceptualised as three Care Stages (CS) representing different degrees of successful toileting, and five Skin Health Levels (SHL) representing different severities of skin problems. Each of the CS and SHL are associated with costs and effects calculated over a lifetime horizon (the model follows a cohort of individuals until they have all died, this provides the average life years accumulated over which health outcomes and costs are calculated) for each of the six population groups. A time cycle length of 2 months (in which individuals transition across groups, CS, and SHL) was chosen, as this was considered long enough to observe an impact of the new intervention and short enough that it is unlikely patients would transition multiple time during the cycle.

### Model structure

The structure of the decision analytic model aims to reflect the T&CC pathway for a heterogeneous population with chronic UI and was determined through interviews with the earlier referred to experts in the field of UI and related care strategies. The three dimensions identified were: 1) a heterogeneous population with changing T&CC needs over time, 2) varying toileting success, and 3) skin health as main care consequence of T&CC strategies.

**Fig 2:**
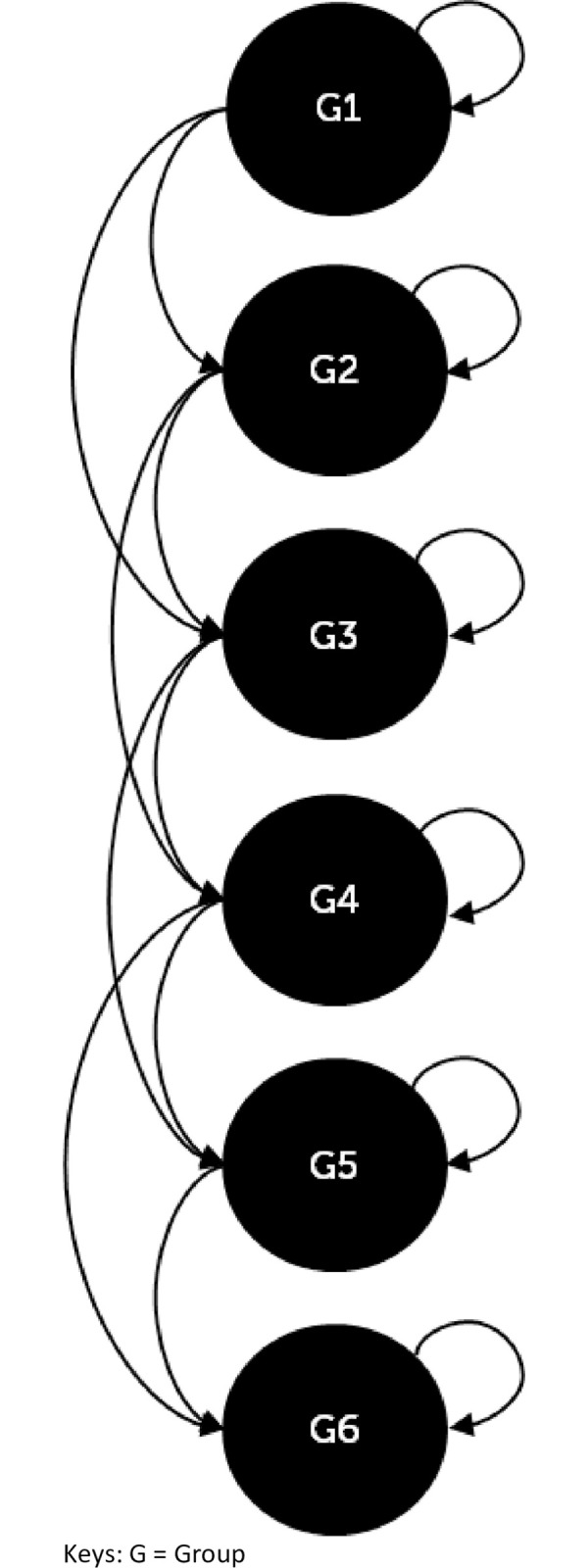
Group transitions.

The natural decline in mobility, dexterity, and cognition of individuals over time resulting in an increased need for assistance with T&CC is represented by the vertical and diagonal arrows in the model (Fig 1). Along these arrows, individuals progress to groups that represent different degrees of disability (see Table 1). The progression is not always sequential since physical and cognitive decline may not occur in parallel. For example, a person with mild confusion may further deteriorate to significant confusion but remains entirely mobile. Fig 2 depicts the possible transitions across the six population groups in this model. When individuals transition to another group, their care stage may remain the same or deteriorate alongside the physical and/or cognitive abilities (vertical and diagonal arrows in Fig 1). The model allows for deterioration but not improvement in disability level. Each of the six groups has a group-specific risk of death.

The different degrees in incontinence severity and the associated toileting success are represented by CS1 to CS3. In CS1 individuals with UI symptoms achieve successful toileting as their main T&CC strategy, whereas in CS3 individuals receive mainly absorbent products. CS2 describes a mix of both strategies. Over time, severity of UI may increase, which may lead to a transition to a less favourable care stage (horizontal arrow pointing to the right). Through adequate care, it may be possible to improve toileting, visualised as the vertical and diagonal arrows pointing to the left. The CS levels are operationalised as absorbency ranges to reflect mild, moderate, and heavy use of absorbent products, defined according to total absorbency of products used over a 24-hour period, with different ranges for average-sized and large residents. Total absorbency is calculated by multiplying the average number of products used by the theoretical absorbency of each product, as derived from the laboratory test described in ISO 11948–1 *(*also known as the *Rothwell value)*. The ISO 11948–1 specifies a method that measures the maximum absorption capacity of the absorbing material in the entire urine-absorbing aid [37]. Experts from Essity provided the cut-off points for each CS (Table 2). S1 and S4 Tables in S4 File outlines the approach taken to arrive at the respective cut-off points.

**Table 2:** Care Stage (CS) definition.

	Care Stage 1	Care Stage 2	Care Stage 3
**Definition**	Mainly successful toileting	Increased containment care	Mainly containment care
**Measurement**	Total absorbency over 24-hours		
**Average residents**	**< 3800**	**3800–5700**	**> 5700**
**Larger residents**	**<4500**	**4500–6750**	**> 6750**

The reassessment of a person’s disability level (group) and incontinence severity (care stage) is represented by the two-monthly model cycle. In this model no excess mortality due to more severe incontinence was assumed.

The third and final dimension of the model accounts for skin health problems, the most direct care consequence when managing UI symptoms. Five levels of skin health were defined with SHL1 indicating the absence of any skin health problems, and SHL5 indicating very severe skin health problems (Table 3). The SHL is assessed by a nurse. Further information on the SHL assessment is provided in S2 File. Every cycle, individuals in each of the three CS are assigned to one of the five SHL. Effectiveness of interventions on SHL are directly captured by the probabilities associated with SHL allocation.

**Table 3:** Skin health levels (SHL).

**Skin Health Level 1**	No skin problems
**Skin Health Level 2**	Mild skin problems
**Skin Health Level 3**	Moderate skin problems
**Skin Health Level 4**	Severe skin problems
**Skin Health Level 5**	Very severe skin problems

### Model inputs

In the section below, we describe the inputs for a residential care population. However, the model allows for inclusion of inputs specific for community-dwelling individuals.

*Flow of individuals between care need groups*. Each model cycle, residents either remain in the same care need group or transition to another group. It is assumed that the probability to transition from one group to another does not depend on the incontinence care received. These probabilities for group transitions due to cognitive impairment and mobility were derived from Davis et al. [38] and Yu et al. [39] and are outlined in the table below (Table 4). If residents transition to another group, 10% were assumed to remain in their original CS and 90% would move to the next worse CS (e.g., 10% of residents who transition from G2-CS2 to G3 remain in CS2 whilst 90% transition to CS3). This reflects the principle that increased disability is likely to affect the ability to toilet successfully when no adjustments to the care are made. All residents in CS3 who transition to another group remain in CS3. The combined transition probabilities of group and CS can be found in S2 and S4 Tables in S4 File. Group-specific mortality probabilities were derived from Allers & Hoffmann [40] and weighted for sex (Table 5).

**Table 4:** Group transition probabilities per 2 months.

Group transition	Probability per cycle	Source
Group 2—Group 2	0.935	[38,39]
Group 2 -> Group 3	0.063	
Group 2 -> Group 4	0.002	
Group 3—Group 3	0.914	
Group 3 -> Group 4	0.064	
Group 3 -> Group 5	0.022	
Group 4—Group 4	0.393	
Group 4 -> Group 5	0.585	
Group 4 -> Group 6	0.022	
Group 5—Group 5	0.978	
Group 5 -> Group 6	0.022	
Group 6—Group 6	1.000	
Group transition–remain in original CS	0.100	Assumption
Group transition–move to CS with less successful toileting (CS2 or CS3)	0.900	Assumption
Group transition–CS3 remain in CS3	1.000	Assumption

**Table 5:** Probability of death per 2-month cycle.

Probability of death–weighted, per cycle									
	*Group 1*			*Group 4*			*Groups 2*, *3*, *5*, *6*		
Age group	Mean	*95% CI*		Mean	*95% CI*		Mean	*95% CI*	
** 65–74 years**	0.069	*0*.*066*	*0*.*071*	0.036	*0*.*033*	*0*.*038*	0.055	*0*.*054*	*0*.*057*
** 75–84 years**	0.064	*0*.*063*	*0*.*066*	0.048	*0*.*047*	*0*.*050*	0.056	*0*.*055*	*0*.*057*
** 85–94 years**	0.069	*0*.*068*	*0*.*071*	0.068	*0*.*067*	*0*.*070*	0.069	*0*.*068*	*0*.*070*
** 95 + years**	0.087	*0*.*082*	*0*.*092*	0.102	*0*.*094*	*0*.*109*	0.092	*0*.*088*	*0*.*097*

Source: **[40]** CI = confidence interval.

The effectiveness of an intervention is determined by the transition probabilities across CS and SHL per group, i.e. new interventions have the ability to change the horizontal transition probabilities. The effectiveness is assumed to remain constant over the remaining lifetime. The effects of care consequences are implemented as rate per cycle per group.

Initial distributions for the groups, CS, and SHL are country-, region-, or provider-dependent.

*Effects*. Each group, CS, SHL, and care consequence is associated with a QoL utility value. As mentioned earlier, in this model, QoL can be estimated with the EQ-5D-5L or the Alzheimer’s disease-related quality of life scale (QoL-AD) [34,36]. The model uses utility decrements which are specific to group, CS, and SHL, providing the opportunity to account for possible interrelations between utility decrements for CS and SHL. This is done by defining a baseline utility per group, for residents in CS1 and SHL1, and when the CS and SHL increase, a disutility is applied to reflect a loss of QoL. Likewise, UI related adverse events, i.e. PU, constipation, UTI and fractures will lead to a temporary disutility.

*Costs*. As health economic guidelines differ across jurisdictions, both direct and indirect costs as well as Out-Of-Pocket (OOP) costs relevant to T&CC are included in the model. Direct costs include technology acquisition costs, labour costs associated with continence care tasks (toileting assistance, product checks, product changes (incl. perineal hygiene), and change of bedlinen with/without clothes changes due to leakage), absorbent product acquisition costs, costs for perineal hygiene products, and costs associated with care consequences. Indirect costs include informal caregiver costs and disposal costs of absorbency products.

Direct costs are estimated based on the resource use and the cost per unit of resource use for each model cycle. Costs for continence care tasks are calculated by multiplying the frequency (varies by type of incontinence care), time needed per task in minutes (Table 6), number of staff required per care task (Table 7), and labour costs per minute (varies per country, Canadian unit costs are in S3 and S4 Tables in S4 File) for the different groups.

**Table 6:** Time spent per continence care task.

Average time spent per continence care task/ per staff, in min.	G2	G3	G4	G5	G6	Source
Product check	3.20	4.10	4.10	4.10	4.10	Essity Hygiene and Health AB, data on file (excerpt in S3. Supplementary data)
Product change (incl. perineal hygiene)	6.60	10.20	10.20	10.20	10.20	
Toileting assistance	9.50	11.70	11.70	11.70	11.70	
Product leakage requiring clothes change and/or bedlinen change	7.40	9.10	9.10	9.10	9.10	
Applying treatment for SHL5	1.00	1.00	1.00	1.00	1.00	Assumption

**Table 7:** Number of staff required per continence care task.

Number of staff required per continence care task	G2	G3	G4	G5	G6	Source
Product checks	1.00	1.00	1.00	1.00	1.00	Assumption
Product change (incl. perineal hygiene, excl. faecal accidents)	1.30	1.30	1.30	1.30	2.00	G2-G5:Essity Hygiene and Health AB, data on file (excerpt in S3. Supplementary data) G6: Expert opinion
Toileting assistance	1.30	1.30	1.30	1.30	2.00	
Product leakage requiring clothes change and/or bedlinen change	1.30	1.30	1.30	1.30	2.00	
Applying treatment for SHL5	1.00	1.00	1.00	1.00	1.00	Assumption

Technology acquisition costs are implemented as average price per person. Absorbent product costs are based on the average number of products used of each type multiplied by their respective prices. Costs for perineal hygiene products were estimated by multiplying the number of applications by the product costs per application. The perineal hygiene regime is assumed to be performed with every change of absorbency product. For costs associated with care consequences, event rates per cycle are multiplied by the respective costs per event. For individuals with skin health problems, we assumed treatment is applied at every product change in addition to the (preventive) perineal hygiene regime and takes on average 1 minute. According to the *Proceedings of the Global IAD Expert Panel* [41] only at the most severe IAD level (red with skin breakdown and infection), which is assumed for SHL5, treatment is applied (e.g., anti-fungal cream, topical antibiotic, anti-inflammatory products) whilst for the other levels the standard perineal hygiene regime is continued. The duration of the skin treatment was set to 7 days [42].

Informal care costs are implemented as percentage of the total continence care provided and can range from 0% to 100%. Thus, the unit costs are estimated as a weighted average of the unit costs for informal caregiving and the unit costs for formal care and multiplied by the resource use in hours of care.

Costs for the disposal of absorbent products are calculated multiplying disposal tariffs per kg of incontinence-associated waste by the total kg of waste produced. Disposal tariffs are based on the volume of the container and number of garbage collections per week.

*Care efficiency and disposal weight*. The total time spent on continence care tasks is estimated in hours and calculated in the same fashion as the costs for continence care tasks but without multiplying the resource use by labour costs. For estimating the amount of waste created due to absorbency product use in kg, the average number of products used of each type is multiplied by their respective dry weight.

## Case study—digital health technology for the Canadian residential care setting

Various T&CC interventions can be evaluated with the *de-novo* model. The first technology to be assessed with the model we developed, and subject of this case study, was the TENA SmartCare Identifi DHT that tracks an individual’s urinary voiding or involuntary leakage to provide insight into patterns of timing and volume. This information helps determine recommended toileting times, the most appropriate absorbent products, and adjustment of care routines for the individual.

### Intervention and comparator

We evaluated the DHT in combination with training on the DHT and current PCCC standards. The DHT consists of a sensor placed in an absorbent product that detects the onset of a wetness event. The sensor is connected through a wireless network with a desktop computer where all voiding and involuntary leakage events and volumes per void / leak during the 72-hour assessment can be viewed. The aim of this automatised digital bladder diary is to support staff with the drafting of a personalised care plan (i.e., individual toileting scheme and choice of absorbent product). The technology has been tested in several nursing homes in the Netherlands [24], Sweden, and Canada. In 2020, the effectiveness of the TENA SmartCare Identifi DHT was studied in a prospective, parallel arm–controlled trial [43]. The trial took place in four units of one Canadian nursing home and evaluated the impact of the DHT (N = 43) versus usual care in the control arm (N = 46) after 8 weeks. The primary outcome was the between-group change in the proportion of residents with a reduction in total absorbency of absorbent products at 8 weeks compared with baseline. Total absorbency was defined as category of the absorbent product (using the TENA® defined absorption levels) multiplied by the number of products used for day, night, and 24 hours. Secondary outcomes included amongst others, AD-QoL, number of wet checks and leakages, and occurrence of IAD. The usual care level of the participating nursing home was identified as mainly conventional continence care (Level 3). The results of this trial constitute a key element of the evidence base driving this economic evaluation.

### Input parameters

For this analysis, the DHT was evaluated in comparison with Level 3 conventional T&CC in G2 to G6 for the Canadian nursing home setting. In this analysis we considered a population with a mean age of 86 and 74% female, as reported in the ARCTICC study. A discount rate of 5% was applied to costs and effects according to Canadian economic evaluation guidelines [32].

Values for input parameters were derived from resident-level data of the two-arm ARCTICC study and published literature wherever possible; otherwise, expert opinion was sought.

*Flow of residents*. Initial distributions for the three CS were derived from the total cohort in the ARCTICC study. Data on group allocation was not collected, therefore, we assumed the same initial CS distributions for G2 to G6. In the absence of Canadian data, the initial group distributions were based on Tabali *et al*. [44], Schäufele *et al*. [45], and Heinze *et al*. [46]. Proportions for different levels of skin health (i.e., redness, rash, skin loss/breakdown, bleeding) reported by Bliss *et al*. [42] in combination with IAD occurrence of the total cohort at baseline in the ARCTICC study were used to estimate the initial SHL distributions. Table 8 outlines the initial distributions for groups, CS, and SHL.

**Table 8:** Input parameters–initial distributions.

Initial distribution	Percentage	Source
Group 2	0.21	[44–46]
Group 3	0.46	
Group 4	0.19	
Group 5	0.09	
Group 6	0.05	
Care Stage 1	0.16	ARCTICC study
Care Stage 2	0.47	
Care Stage 3	0.37	
Skin Health Level 1	0.89	ARCTICC study, [42]
Skin Health Level 2	0.07	
Skin Health Level 3	0.02	
Skin Health Level 4	0.02	
Skin Health Level 5	0.01	

*Effectiveness of the conventional care and the DHT*. CS transition probabilities were based on the ARCTICC study. The probabilities were calculated using the distribution of residents across CS at baseline and at week 8 for the intervention and control arm (Table 9). In the ARCTICC study, no data were collected to differentiate between groups. We therefore assumed the same CS transition probabilities apply for G2 to G6.

**Table 9:** Input parameters–effectiveness–transition probabilities between care stages.

Care Stage transition	Conventional care	DHT	Source
CS1 → CS1	0.38	0.25	ARCTICC study
CS1 → CS2	0.63	0.75	
CS2 → CS2	0.45	0.68	
CS2 → CS3	0.45	0.09	
CS2 → CS1	0.10	0.23	
CS3 → CS3	0.44	0.59	
CS3 → CS2	0.50	0.35	
CS3 → CS1	0.06	0.06	

The probability for developing skin health problems (SHL1-5) in each of the three CS was derived from resident-level data from the two arms in the ARCTICC study in combination with proportions found by Bliss *et al*. [42] (Table 10). A Difference-in-Difference (DiD) approach was applied to changes in skin health problems (as well as the number of leakages, and number of checks) to control for significant baseline differences that were found for number of absorbent products used, total absorbency, and number of product leakages.

**Table 10:** Input parameters–effectiveness SHL–transition probabilities per cycle.

	G2—G6	SHL 1	SHL 2	SHL 3	SHL 4	SHL 5	Source
DHT	**CS1**	1.000	0.000	0.000	0.000	0.000	ARCTICC study, [42]
	**CS2**	0.972	0.018	0.004	0.004	0.003	
	**CS3**	1.000	0.000	0.000	0.000	0.000	
Conventional care	**CS1**	1.000	0.000	0.000	0.000	0.000	
	**CS2**	0.972	0.018	0.004	0.004	0.003	
	**CS3**	0.875	0.075	0.016	0.016	0.013	

T&CC related adverse care consequences included fractures resulting from falls associated with toileting, UTI, constipation, and PU. The incidence rate per cycle for fractures was calculated based on the 6-month incidence of falls measured in community-dwelling women receiving home support [47] due to lack of literature for the nursing home setting; combined with the proportion of falls related to toileting in institutions [48] (between 20% and 50%) and the estimated percentage of falls resulting in a fracture (5.7%) [26]. Given the very limited mobility of individuals in G5 and G6, an incidence rate of 0.00 was assumed for fractures. For UTI a 2-month incidence rate of 0.084 and for constipation a rate of 0.098 was derived based on Omli *et al*. [15] Incidence rates for PU category I-IV were derived from Makai *et al*. [49] (see S4 Table in S4 File for a definition of the PU categories). No data were available to estimate the impact of the DHT on fractures, UTI, and PU. We therefore used estimates on reductions in care consequences from one expert (S4 and S5 Tables in S4 File). Incidence rates for care consequences are outlined in Table 11.

**Table 11:** Input parameters—care consequences–probability per cycle.

Care consequence	Conventional care	DHT*	Source
Toileting related fracture G2-G4	0.0027	0.0026	[26,47,48]
Toileting related fracture G5-G6	0.0000	0.0000	Assumption
UTI	0.0577	0.0543	[50]
Constipation	0.0894	0.0787	[15]
PU category 1	0.2940	0.2917	[49]
PU category 2	0.0680	0.0675	
PU category 3	0.0074	0.0073	
PU category 4	0.0154	0.0153	

*Expert opinion (n = 1), G = Group, PU = Pressure Ulcer, UTI = Urinary Tract Infection.

*Costs*. All costs are reported in CAD 2020 with prices corrected for inflation where necessary. For continence care costs, the resource use is outlined in S4 and S6 Tables in S4 File for DHT and in S4 and S7 Tables in S4 File for conventional care. Labour costs were derived from Ba’Pham *et al*. [51] Costs for absorbent products for each CS were based on resident-level data from the ARCTICC study and prices per product type from Essity. Unit costs for perineal hygiene products for maintaining skin health were based on Palese & Carniel [22]. Higher costs were assigned to the DHT group, assuming the use of better hygiene products following the F2F PCCC training. The number of absorbent products for calculating costs for product disposal and waste in kg were based on resident-level data from the ARCTICC study. The tariff for disposal was based on grey literature for a region in Germany due to the lack of data. Incontinence-associated waste was assumed to account for 54% [52] of the entire residual waste of an average nursing home. Unit costs for treatment of SHL5 were derived from Raepsaet *et al*. [53]. For other care consequences, costs were derived from Meerding *et al*. [54] (fractures), Hakkaart-van-Roijen *et al*. [31] (UTI), Frank *et al*. [55] (constipation), and Bennett *et al*. [56] (PU). Acquisition costs for the DHT including software and assessment briefs with sensors for one assessment per resident per year were provided by Essity. Unit costs can be found in the S3 and S4 Tables in S4 File.

*Health benefits*. EQ-5D utilities to estimate QALYs were not available in the ARCTICC study, and therefore derived from the literature. For G1, the utility score was based on findings from Poder *et al*. [57]. To estimate QoL for the other groups, utility decrements were calculated based on the EQ-5D-5L utility values reported by Easton *et al*. [58] for five different dependency levels of nursing home residents in Australia. The percentage decrease between G1 and the other groups were applied to the EQ-5D-5L utility value reported by Poder *et al*. A similar approach was applied to determine utility decrements for CS2 and CS3 (for CS1 a utility decrement of 0 was assumed). Proxy-reported AD-QoL was collected in the ARCTICC study, therefore, QoL data were available for the three CS. We calculated the absolute differences between the AD-QoL value for CS1 and the other two CS. These (absolute) CS decrements were applied to the AD-QoL value for G1 to arrive at the relative CS decrements, which we applied to the EQ-5D-5L value for G1. The AD-QoL value for G1 was derived from Chan *et al*. [59]. Since AD-QoL in the ARCTICC study was collected with the 13-item AD-QoL questionnaire and the AD-QoL from Chan *et al*. with the 15-item AD-QoL questionnaire, we re-scaled the values for G1, CS2, and CS3 to between 0 and 1. Utility decrements for SHL2 to SHL5 were derived from literature. SHL1 represents no skin problems, therefore a utility decrement of 0.00 was assumed. Considering the relatively subtle increase in severity from SHL2 to SHL3 we assumed the same utility decrement for both levels. For SHL5 we assumed the same utility decrement as for PU. SHL4 utility decrement was estimated as the average of SHL3 and SHL5. All utility decrements can be found in Table 12.

**Table 12:** Group utility and utility decrements.

Utility	Value	Source
G1	0.80	[57]
G2	0.70	[58]
G3	0.64	
G4	0.54	
G5	0.30	
G6	0.30	
**Utility decrements**		
CS1	0.000	
CS2	-0.014	Estimated
CS3	-0.030	Estimated
SHL1	0.000	
SHL2	-0.033	[60]
SHL3	-0.033	Assumption same as SHL2
SHL4	-0.035	Average of SHL3 and SHL5
SHL5	-0.037	Assumption same as PU
Fractures*	-0.121	[61]
UTI	-0.090	[62]
Constipation	-0.030	[63]
PU Cat. I	0.000	[51]
PU Cat. II	-0.037	
PU Cat. III	-0.037	
PU Cat. IV	-0.037	

CS = Care Stage, G = Group, PU = Pressure Ulcer, SHL = Skin Health Level, UTI = Urinary Tract Infection.

*Resulting from falls associated with toileting.

### Assessment of uncertainty

The influence of parameter uncertainty on the results was assessed by one-way sensitivity analysis (OWSA) and probabilistic sensitivity analysis (PSA). In the OWSA, the lower and higher bound of the confidence interval (CI) were used for each parameter. If not reported, CIs were estimated based on the mean and standard error (SE). A PSA was performed to quantify the level of confidence in the results of the analysis in relation to uncertainty in the model inputs. In the PSA, the values of all parameters are simultaneously varied. A beta distribution with a standard error equal to 20% of the mean was applied in the PSA for all parameters limited to the interval of 0 and 1, such as the average number of skin health treatment applications and utility decrements for care consequences except for SHL. A Dirichlet distribution was applied to all parameters with interdependent probabilities including initial distributions and transition probabilities for the groups, CS, and SHL. For costing parameters and resource use random values were drawn from a Gamma distribution with a standard error equal to 20% of the mean. For age, mortality, and utility decrements for the groups, CS, and SHL a normal distribution was applied. Values for parameter cycle length, time horizon, discount rate, CS1 utility decrement and SHL1 utility decrement as well as the number of staff required for continence care tasks (G2-G3) were not varied.

### Results of case study

#### Effects

**Fig 3:**
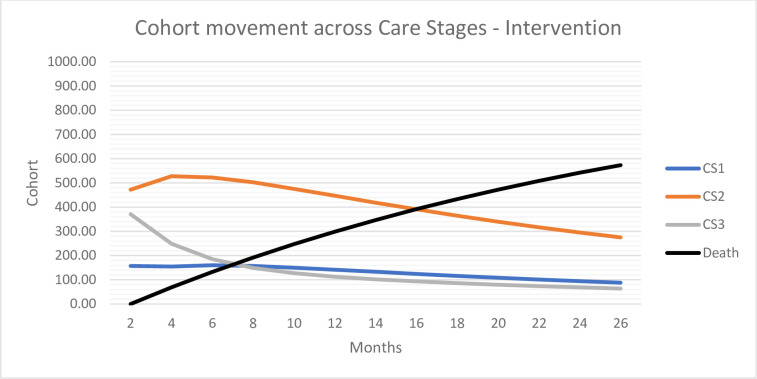
Cohort movement across care stages–DHT.

**Fig 4:**
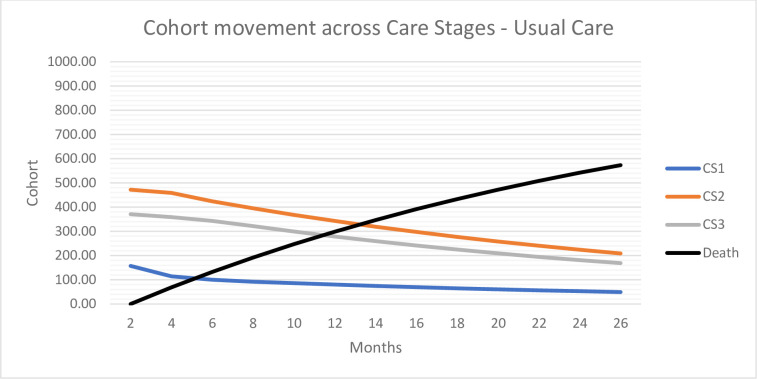
Cohort movement across care stages—conventional T&CC.

In both arms, 57% of the individuals were alive after 2 years (cycle 12) and on average the life years accumulated by the individuals were 2.34. The number of residents per CS and SHL over time determined the effectiveness of the DHT. The DHT was more effective in improving toileting success than conventional T&CC (Level 3). After 2 years, 21% of the surviving individuals managed with the help of the DHT received mainly toileting as T&CC strategy (CS1), and thus had fewer incontinence-related care consequences; 64% received a mix of toileting and containment (CS2); and 15% received mainly containment care (CS3) (Fig 3). In comparison, with conventional care 12% of residents were allocated to CS1, 49% to CS2 and 40% to CS3 (Fig 4). DHT was also more effective in maintaining skin health. After 2 years, 98% of the individuals managed with the DHT had no skin health problems (SHL1) compared with 94% of the individuals managed with conventional care. S1 Fig shows the cohort movement over time for the DHT and conventional care.

More QALYs were accumulated per person with the DHT than with conventional care over a lifetime horizon (Table 13). The incremental gain due to more successful toileting (Row *Care Stage* in Table 13) had the largest impact on total QALY gain of 0.015. A gain in QALYs (0.003) was estimated for skin health with the DHT compared with conventional care. For other care consequences, a gain of 0.002 was achieved compared with conventional care.

**Table 13:** Total QALYs and incremental QALYs (lifetime horizon, discounted).

		Conventional T&CC	DHT	Difference
**Total QALYs**	** **	0.871	0.886	**0.015**
**Care Stage**		0.906	0.916	**0.010**
**SHL**		-0.0045*	-0.0015*	**0.003**
**Care Consequences**		-0.030	-0.028	**0.002**

DHT = Digital Health Technology, SHL = Skin Health Level, T&CC = Toileting & Containment Care.

* Four decimals presented to avoid rounding difficulties.

Over the residents’ average remaining lifespan of 2.34 years, a reduction of 36 hours spent on continence care tasks per resident with the DHT compared with conventional care. Whilst time spent on continence care remained the same in G2 and G4, it reduced in G3, G5 and G6 with the DHT (see Table 14). The DHT also reduced the amount of disposal of absorbent products (using dry product weight) by 32kg per resident over the 2.34 life years, a relative reduction of about 14% (see Table 15).

**Table 14:** Care efficiency, in hours per resident over lifetime horizon.

	Conventional T&CC	DHT	Difference
**Total Hours**	831	794	**-36**
**Group 2**	68	67	**0**
**Group 3**	219	216	**-3**
**Group 4**	36	35	**0**
**Group 5**	347	339	**-7**
**Group 6**	161	136	**-25**

DHT = Digital Health Technology, T&CC = Toileting & Containment Care.

Note: Numbers are rounded.

**Table 15:** Disposal, in kg per resident over lifetime horizon.

	Conventional T&CC	DHT	Difference
**Total Kg**	219	187	**-32**
**Group 2**	25	21	**-3**
**Group 3**	57	49	**-8**
**Group 4**	9	8	**-1**
**Group 5**	90	77	**-14**
**Group 6**	38	32	**-6**

DHT = Digital Health Technology, T&CC = Toileting & Containment Care.

Note: Numbers are rounded.

### Costs

Total costs (discounted) were lower for DHT than for conventional care ($42,212 vs. $43,679, respectively) with cost savings of $1,467 per resident over a lifetime horizon. Most cost savings were achieved with lower costs for absorbent products. With the DHT, costs for absorbent products were $3,793 per resident over a lifetime horizon compared with $5,172 with conventional care resulting in cost savings of $1,379, or a reduction in absorbent product costs of 27%. The acquisition cost of the DHT accumulated to $546 per resident over a lifetime horizon (Table 16). Labour costs associated with continence care resulted in $15,938 per resident with the DHT in comparison to $16,508 with conventional care resulting in savings of $570 per resident over a lifetime horizon. Perineal hygiene product costs per resident were higher with the DHT ($345) than with conventional care ($161). The costs for the disposal of absorbent products were reduced by $7 per resident with the DHT over a lifetime horizon. Costs for care consequences resulted in cost savings of $240 per resident with the DHT over a lifetime horizon. Costs for skin health problems were negligible for both current and new care.

**Table 16:** Total costs and incremental costs (lifetime horizon, discounted).

	Conventional T&CC	DHT	Difference
**Total **	$ 43,679	$ 42,212	**-$ 1,467**
**Technology implementation**	$ -	$ 546	**$ 546**
**Labour costs associated with continence care tasks**	$ 16,508	$ 15,938	**-$ 570**
**Absorbent product**	$ 5,172	$ 3,793	**-$ 1,379**
**Perineal hygiene product use**	$ 161	$ 345	**$ 184**
**Product disposal **	$ 50	$ 42	**-$ 7**
**Care consequences**	$ 21,787	$ 21,547	**-$ 240**
**SHL5 **	$ 1	$ 0	**-$ 1**

DHT = Digital Health Technology, SHL = Skin Health Level, T&CC = Toileting & Containment Care.

Note: Numbers are rounded.

### Cost-effectiveness

Cost-effectiveness of the DHT compared with conventional care was evaluated for a lifetime horizon. Given the gain in QALYs (0.015) and the reduction in costs per resident (-$1,467), the DHT is considered dominant and, therefore, preferred over conventional care (from a CE perspective).

### Uncertainty analysis

**Fig 5:**
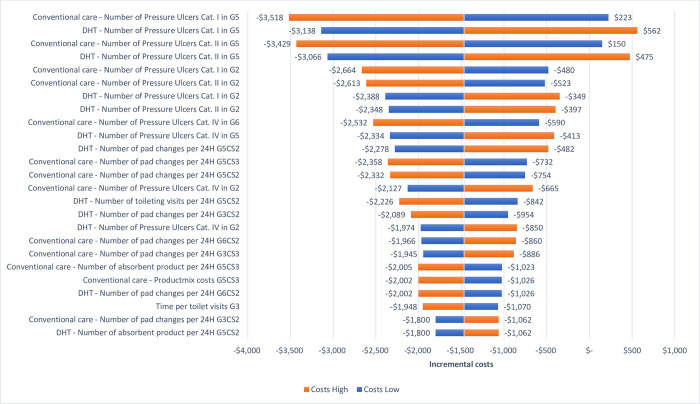
OWSA—incremental costs.

**Fig 6:**
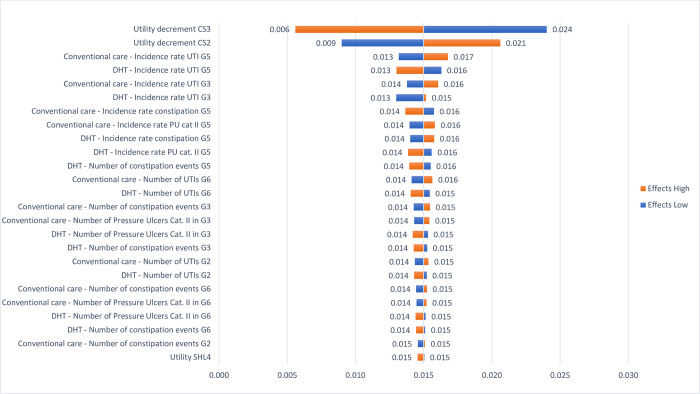
OWSA—incremental effects.

Results from the OWSA are presented in Figs 5 and 6 showing the impact of influential parameters on incremental costs and effects, respectively. The OWSA shows that the parameter *number of pressure ulcers*, in both DHT and conventional care, have the largest impact on incremental costs ranging from cost savings of $3,518 to additional costs of $562 followed by the *number of product changes* in G3, G5, and G6 in CS2 and CS3 with incremental costs ranging between -$2,358 and -$482. Incremental effects are impacted mainly by changes in utility values for CS3 and CS2 and decrease to 0.006 incremental effects with a higher CS3 utility decrement and increase to 0.024 with a lower CS3 utility decrement. For CS2 the impact of varying the base case utility value is less pronounced than for CS3, with incremental effects of 0.009 and 0.021 with a lower and higher utility decrement, respectively.

**Fig 7:**
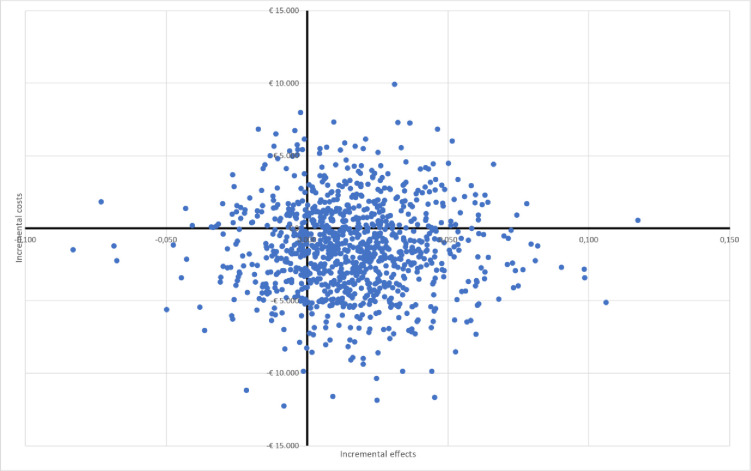
Cost-effectiveness plane.

**Fig 8:**
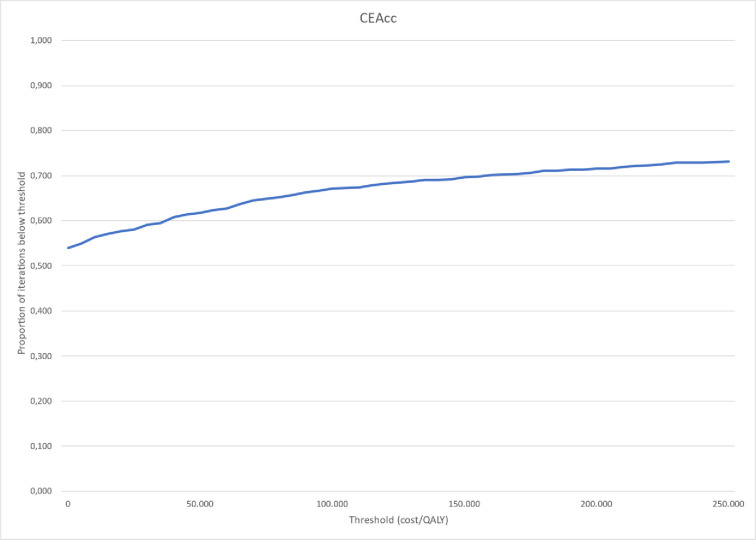
Cost-effectiveness acceptability curve (CEAcc).

The results of the PSA show dominance of the DHT over conventional care with average incremental costs of -$1,433 (-$1,467 deterministic estimate) and average incremental effects of 0.016 (0.015 deterministic estimate). Fig 7 shows the probability that DHT is dominant, with 52% of the iterations resulting in incremental costs and effects in the bottom right quadrant of the CE Plane, where the DHT yields more effects and reduces costs and therefore dominates conventional care. About 16% fall within the bottom left quadrant of the CE Plane indicating cost savings with the DHT and QALY losses compared with conventional care. The implication of results in this quadrant is that the achieved cost savings can be used to invest in other health technologies that bring additional QALYs. In 24% of the iterations, the ICER falls within the top right quadrant for which governmental bodies and regional decision-makers commonly define a willingness-to-pay threshold for every QALY gained. Depending on that threshold, the intervention may or may not be deemed cost-effective. The other 8% of iterations fall within the top left quadrant in which the comparator dominates the intervention. Fig 8 depicts the cost-effectiveness acceptability curve for the DHT at a range of accepted costs per QALY gained showing that at a threshold of $50,000 the probability of the DHT being cost-effective is approximately 62%. Depending on the threshold ICER, the probability of DHT being cost-effective varies between 55% and 72%.

## Discussion

This cost-effectiveness study showed that the DHT is more effective and less costly than conventional care. The DHT resulted in improved toileting, fewer incontinence-related care consequences, a higher QoL and less waste of absorbent products than conventional care. Costs were reduced by $1,467 per resident over a lifetime duration with the DHT compared with conventional care. In the probabilistic sensitivity analysis, 52% of all iterations confirmed the dominant result of the deterministic analysis. A further 24% of all iterations resulted in more QALYs and costs for the DHT. Given a willingness-to-pay threshold of $50,000 the probability of the DHT being cost-effective is 62%.

### Existing literature comparison

To our knowledge, no economic evaluation exists for toileting and containment care strategies in nursing homes. The cost-effectiveness study by Holtzer-Goor *et al*. [26] provides some insights into costs and effects due to improved incontinence severity with a PCCC strategy. Holtzer-Goor *et al*. found a QALY gain of 0.005 in combination with cost-savings for introducing continence nurse specialists in the care pathway of older community-dwelling adults. These findings align with our findings of a person-centred T&CC intervention dominating conventional T&CC. The small incremental QALY gains reported by Holtzer-Goor *et al*. and in this study may be due to the heterogeneous population group, where some, but not all, patients improve due to the new care for incontinence, and the measurement of QoL with a generic questionnaire that may not sufficiently elicit condition-specific changes in QoL.

In addition, one Australian [23] and two European studies [22,24] reported on the beneficial effects on health and costs with personalised T&CC strategies. Yu *et al*. [23], who studied a similar digital health technology in Australia, reported a decrease in incontinence episodes and improvements in successful toileting compared with standard care. These findings align with our reported outcomes of 21% of surviving residents remaining in CS1 after two years, indicating improvements in successful toileting with the DHT, compared with 12% of residents with conventional care.

A reduction of IAD prevalence was achieved in the study conducted by Palese *et al*. [22] after introducing new absorbent product types and a specialist incontinence nurse. In our study, we found a small effect on skin health. Palese *et al*. further reported a decrease of 61% in the number of absorbent products and associated cost reductions of 56%. In our study, we found a reduction of 14% in total absorbency (based on the absorbent product capacity measure expressed as a Rothwell value measured according to the methods set out in the ISO 11948–1 standard, multiplied by the number of pieces used) accompanied by a cost reduction of 27% for absorbent products with the DHT compared with conventional care. Similar figures to our study were reported by van Baalen & Brocken [24] on the impact of the same DHT in two Dutch nursing homes, reporting a reduction of 14% and 28% in total absorbency in the respective facilities.

The reduction in disposal of absorbent products found in this study (14%) is lower than reported by Palese *et al*. (75%) but aligns closely with the reduction observed in one of the two Dutch nursing homes reported on by van Baalen & Brocken, one nursing home reported a reduction of 19% and the other of 49%. The large difference in disposal reduction between the study by Palese *et al*. and our study may be explained by the introduction of a new brand of absorbent products in the Palese study whereas our intervention focused on the optimisation of already available absorbent product types. Reductions in disposal costs due to a person-centred continence care approach could not be found in literature. In this study we estimated a reduction in disposal costs of $7 per resident over a lifetime with the DHT. The reduction of disposal costs is likely to be an underestimate of the true cost savings since dry product weight was used for the calculations due to the lack of data on the weight of used product requiring disposal as well as the lack of robust costing data.

### Strengths and limitations

This *de-novo* cost-effectiveness model was developed with input from three countries (Canada, Germany, The Netherlands) to adequately present the different mechanisms that play a role in the management of UI symptoms. The effectiveness of T&CC interventions is captured through 1) the transitions to CS with greater toileting success and, therefore, higher QoL, and 2) through higher probabilities for good skin health, and therefore QoL gains. Additionally, since the resource use in the model is determined per care strategies and not set per CS, interventions that aim at personalised continence care can achieve cost savings even in CS associated with increased containment by optimizing the care for individuals. Therefore, PCCC interventions that focus on improving skin health can also be assessed with this model. Furthermore, our model differentiates between the older adult population with incontinence symptoms, an important aspect confirmed by DuBeau *et al*. [8] who showed a significant association between severity of incontinence symptoms, QoL, and mobility and cognition using the Social Engagement Scale (SocE). For example, in individuals who could still transfer with assistance, although severely cognitively impaired, the severity of incontinence symptoms was significantly associated with changes in QoL. However, this was not shown for residents who were unable to transfer (whilst severely cognitively impaired). In contrast to existing economic models, our model allows for such subgroup analyses.

The comprehensiveness of the model differentiating the heterogeneous population of older adults with incontinence comes at a trade-off for data. Differences in the effectiveness of the intervention could not be established for the various population groups since these data were not collected in the ARCTICC study. In addition, due to the lack of EQ-5D-5L data in the ARCTICC study, utility decrements were derived from the literature and converted from the AD-QoL. Ideally, group, CS and SHL utilities would have been measured in the trial, to ensure the validity of the estimates to the study population and to account for interrelations between decrements. The recently developed mapping algorithm [64] could assist in transforming AD-QoL values to EQ-5D utility values in the absence of EQ-5D measurements, but would also require trial data that distinguish group, CS and SHL. The QALY gains in this analysis are solely driven by utilities, and the utility values have the largest impact in the OWSA on incremental effects.

In this analysis, the effectiveness, represented as transitions to and from the CS, was based on the total absorbency operationalised by the number of products used multiplied by the Rothwell value of the respective absorbent product. The ISO 11948–1 method measures the maximum theoretical absorption capacity of the absorbing material in the entire absorbent product. Measuring the weight of used product is the preferred measure of incontinence severity, but in everyday practice this method is labour intensive and not widely used [10,23,65]. Since the primary source of effectiveness evidence was based on the ARCTICC study, using the ISO 11948–1 method was the best available proxy to measure incontinence severity.

Finally, a limitation might be that it is uncertain to what extend projected cost savings would materialise when implementing the DHT in daily practice. If the hours saved on continence care are small within a certain residential care facility, it might be difficult to reduce the number of staff hours to the optimal number of staff hours (for instance due to permanent and long-term contracts). However, if each week a few hours are saved, that time could be used for other care tasks that may in turn improve quality of life or reduce certain costs. Thus, it would be of interest to closely monitor the implementation of DHT in practice and compare observed cost savings to the modelled outcomes.

### Conclusion and future research

This *de-novo* model allows for the assessment of digital health technologies that are used to develop person-centred T&CC strategies in the total older resident population with UI in both the residential long-term care and home care setting. Person-centredness is emphasised by the stratification of the population by the two main properties associated with toileting success, cognition and mobility, and by adding skin health as another dimension for people for whom toileting is becoming less successful. This model, therefore, represents a more complete picture of benefits achieved with DHT-assisted person-centred T&CC strategies which go beyond toileting success only and, thus, allow for the assessment of a range of future interventions on the toileting and containment care pathway relevant to individuals, care providers, payers, and policy makers alike.

The dominating effect of the DHT assessed in our case study over conventional care confirms earlier studies on beneficial effects of person-centred continence management.

Future studies should focus on exploring further the preferences of dependent and independent individuals with chronic incontinence symptoms as well as formal and informal caregivers and stakeholders on person-centred continence care with support of digital health technologies to crystallise further improvements of this first model framework on the toileting and containment care pathway.

## Supporting information

S1 Fig (PDF)

S1 File Toileting person-centeredness assessment nursing homes. (PDF)

S2 File Skin Health Assessment Measurement. (PDF)

S3 File Time demand for carers of continence care episodes. (PDF)

S4 File (PDF)
