# Supplementary figures and images for: Benefits of a digital health technology for older nursing home residents. A de-novo cost-effectiveness model for digital health technologies to aid in the assessment of toileting and containment care needs
Source: PLoS One. 2024 Jan 2;19(1):e0295846. doi: 10.1371/journal.pone.0295846 (PMC10760782; doi:10.1371/journal.pone.0295846)

Figure S1 Cohort movement across SHL

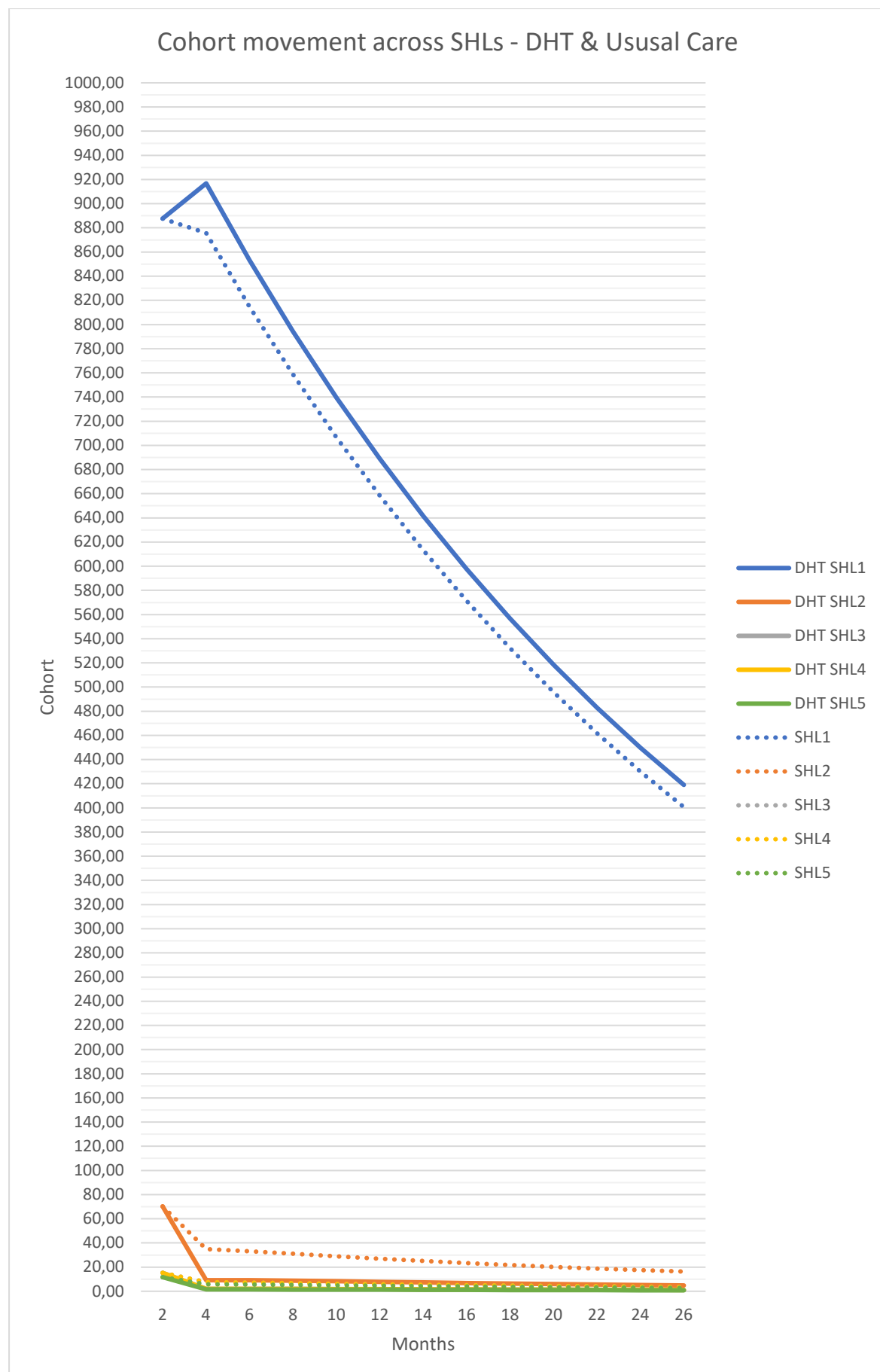

Supplement: S1 Fig — (PDF) [file pone.0295846.s001.pdf]
