# Supplementary material for: Benefits of a digital health technology for older nursing home residents. A de-novo cost-effectiveness model for digital health technologies to aid in the assessment of toileting and containment care needs
Source: PLoS One. 2024 Jan 2;19(1):e0295846. doi: 10.1371/journal.pone.0295846 (PMC10760782; doi:10.1371/journal.pone.0295846)
Supplement: S1 File — (PDF) [file pone.0295846.s002.pdf]

Assessments for use in continence care economic model and SmartCare Change Indicator Clinical Trials

(Reprinted with permission from Essity Hygiene and Health AB)

21 December 2020

1. *Baseline assessment of the level of PCC in a Residential Care Facility*

We expect the benefits of the Change Indicator to vary according to a Residential Care Facility's level of person-centredness of continence care. In order to analyse results by this criterion we need a baseline assessment. As there is no accepted set of assessment criteria, we propose the following, and recommend asking the investigators and the expert panel members whether they feel this method is appropriate.

Scoring system

We propose a 3-tier classification of RCFs into:

- Largely person-centred continence care (PCCC);
- Moderately PCCC;
- Largely conventional continence care

A scoring system will assign one point for each of the six individual PCCC practices listed below and the tiers will be allocated as follows:

- 5 or 6 points: largely PCCC;
- 3 or 4 points: moderately PCCC;
- 0/1 or 2 point: largely conventional continence care.

Individual PCCC practices

Ask senior staff to assess the practice in their facility by answering the following

*Which of the following two statements in each of six areas of continence and personal hygiene care best describes the practice in your nursing home? Please consider the care provided to the **majority** of residents.*

Please place a tick in the box next to either the Person-Centred Continence Care or the Traditional Continence Care practice

1 - INDIVIDUALISED TOILETING SCHEDULE VS. SAME SCHEDULE

	PCC			Traditional
☐	Toileting schedule is based on **individual needs for all/most residents** *(i.e. have different timings for toileting for each resident based on their requirements)*	**versus**	☐	Toileting schedule is the **same for all/most residents** *(i.e. have same timings for toileting for all/most residents such as after meals, before bedtime, etc.)*

2 - INDIVIDUALISED PRODUCT CHANGING SCHEDULE VS. SAME FIXED SCHEDULE

	PCC			Traditional
☐	Changing of incontinence products is organised **individually for all/most residents** *(i.e. tailored to individual needs)*	**versus**	☐	Changing of incontinence products is organised according to the **same fixed schedule for all/most residents** *(i.e. not tailored to individual needs)*

3 - INDIVIDUALISED VS. STANDARDIZED PRODUCT CHOICE

	PCC			Traditional
☐	The main criteria for choosing the type of incontinence product (for example belted product or pants versus all-in-one) are based on **the individual needs** of each resident	**versus**	☐	The main criteria for choosing incontinence products are based on **commonly defined care routines and/or needs** in our nursing home (i.e. not based on individual needs)

4 - FREQUENCY OF CLEANSING AT PAD CHANGE

	PCC			Traditional
☐	The skin in the perineal area of care dependent residents with incontinence is always / mostly **cleansed at every pad change**	**versus**	☐	The skin in the perineal area of care dependent residents with incontinence is **rarely cleansed at pad change**

5 – GENTLE NO-RINSE PRODUCT VS TRADITIONAL PRODUCT CHOICE FOR CLEANSING OF PERINEAL SKIN

	PCC			Traditional
☐	The skin in the perineal area of care dependent residents with incontinence is **cleansed with gentle no-rinse wash cream / mousse**	versus	☐	The skin in the perineal area of care dependent persons with incontinence is **cleansed with regular soap and water**

6- HOLISTIC CONTINENCE CARE VS. FOCUS ON URINE/FAECES CONTAINMENT

	PCC			Traditional
☐	Continence care is defined as including **other factors** than just containment of urine/faeces (e.g. quality of sleep, dignity and skin health)	versus	☐	Continence care is defined as containment of urine/faeces
