# Supplementary material for: Benefits of a digital health technology for older nursing home residents. A de-novo cost-effectiveness model for digital health technologies to aid in the assessment of toileting and containment care needs
Source: PLoS One. 2024 Jan 2;19(1):e0295846. doi: 10.1371/journal.pone.0295846 (PMC10760782; doi:10.1371/journal.pone.0295846)
Supplement: S2 File — (PDF) [file pone.0295846.s003.pdf]

Skin Health Assessment Measurement – Intermittent

(Reprinted with permission from Essity Hygiene and Health AB)

The health economic model described in the main section measures skin health as the principal clinical outcome promoted by good continence care. Poor continence care is known to be associated with a higher incidence of Incontinence Associated Dermatitis (IAD), for which gradations in severity and assessment tools have been described by Clarke-O'Neill (2015) and Van den Bussche et al (2018), leveraging the work of Borchert et al (2010). Relatively few people with incontinence develop the more severe end of the spectrum of skin lesions represented by IAD, with a much greater number experiencing less severe incontinence-induced skin lesions. However, there is no satisfactory assessment tool described in the literature to measure their degree of severity. A five-stage skin health model and assessment tool were therefore developed by Essity for use in clinical trials in preparation on its Digital Health Technology products, and the five stages of skin health were included in the economic model described in the main section.
The assessment tool takes the form of a simple card to be filled in by the research nurse, capturing the severity, extent and location of individual lesions within the area of skin normally covered by an absorbent pad, followed by an overall judgement of severity. The tool is reproduced below.

Aim:

- *Assess skin health at admission*
- *Follow progress of skin problems*
- *Compare skin status over time*

<u>Instruction to fill in the observation form:</u>

A) *Record 3 measurements by filling in 3 observational questions:*

1. *Visual observation by research nurse at start of baseline measurement*
2. *Visual observation by research nurse at midway of trial at 4 weeks*
3. *Visual observation by research nurse at end of trial at 8 weeks*

A.1) *Please look at the skin within the pad area and tick if any of the following issues are present (you may tick more than one box) and indicate where on the skin it is present by circling the number(s) from figure 1. in the box named Location.*

	Normal skin characteristics/temporary marks	Location
	- Please tick more than one box if necessary	*- Circle number(s) for place of issue*
☐	Permanent marks e.g. birth marks, depigmentation (white patches)	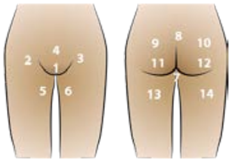
☐	Areas of purple/mauve/discoloration which blanch on pressing	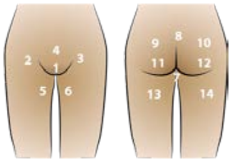
☐	Pad/pants crease marks (temporary pink marks)	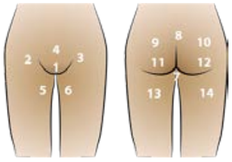
☐	None of the problems above.	

A.2) *Please look at the skin within the pad area and answer the questions about grading of the skin injury, the size and places on the body.*

	Grading	Size	Location
	- Tick box of grading	*- Tick box of size*	*- Circle number for place of rash*
**1**			
☐	**NO** REDNESS. No abnormalities to the skin. 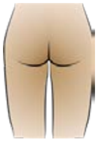		
**2**			
☐	PERSISTENT **REDNESS**. A variety of tones of redness may be present. In persons with darker skin tones, the skin may show paler or darker or purple compared to normal.	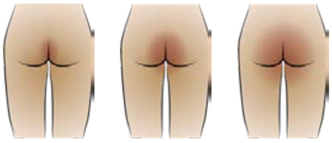 ☐ ☐ ☐	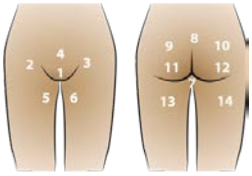
**3**			
☐	PERSISTENT REDNESS WITH SIGNS OF **SATELLITE LESIONS**. As above plus signs of lesions are present, which are abnormal tissue changes.	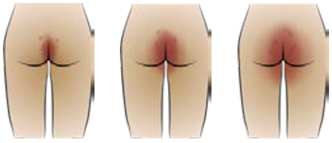 ☐ ☐ ☐	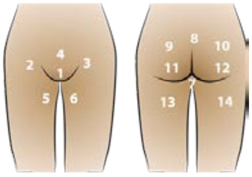
**4**			
☐	PERSISTENT REDNESS WITH SIGNS OF SATELLITE LESIONS AND **SKIN LOSS**. As above plus skin loss may present as skin erosion.	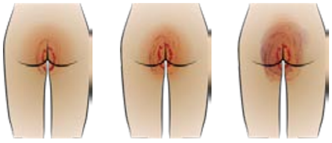 ☐ ☐ ☐	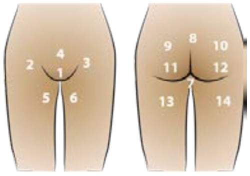
**5**			
☐	PERSISTENT REDNESS WITH SIGNS OF SATELLITE LESIONS AND SKIN LOSS. PLUS SIGNS OF **INFECTION**. As above but signs of infection are present, with areas of white or pustules surrounding the lesion.	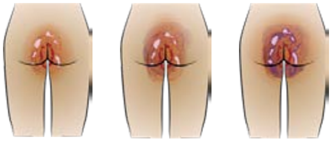 ☐ ☐ ☐	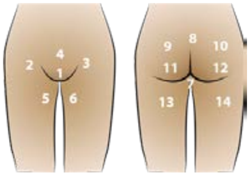

A.3) *Taking everything into consideration - the grading of the skin injury, the size and the number of places on the body - how would you rate the severity of the skin problem(s) overall?*

Overall assessment	
Tick one box	
**None**	☐
**Mild**	☐
**Moderate**	☐
**Severe**	☐
**Very Severe**	☐

Reference: Place on the body:

Figure 1. Visualization where skin issues can appear

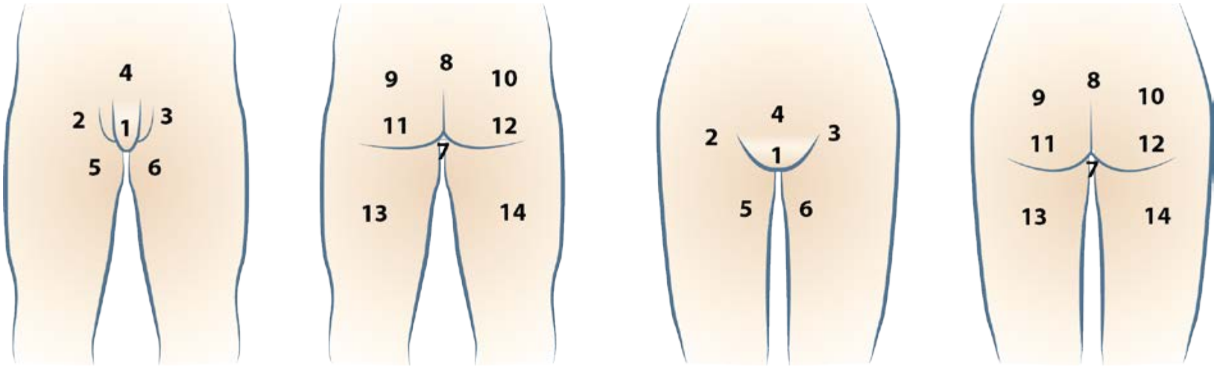

1 Genitalia (labia/scrotum)

2 Right groin fold (crease between genitalia and thigh)

3 Left groin fold (crease between genitalia and thigh)

4 Lower abdomen, suprapubic

5 Right inner thigh

6 Left inner thigh

7 Perianal skin

8 Gluteal fold (crease between buttocks)

9 Left upper buttock

10 Right upper buttock

11 Left lower buttock

12 Right lower buttock

13 Left posterior thigh

14 Right posterior thigh

Reference: Level of redness

Rash/level of redness

Examples of skin colour:
In light skin tone

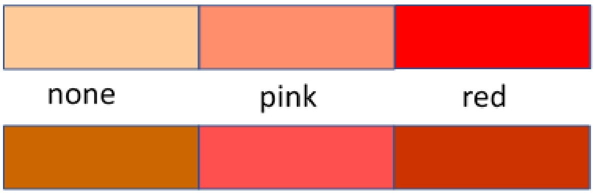

In dark skin tone

References

Sinead Clarke-O'Neill, Anne Farbrot, Marie-Louise Lagerstedt Eidrup, Alan Cottenden, Mandy Fader. Is it Feasible to Use Incontinence-Associated Dermatitis Assessment Tools in Routine Clinical Practice in the Long-term Care Setting? J Wound Ostomy Continence Nurs, Jul-Aug 2015;42(4):379-88.

Kathleen Borchert, Donna Z Bliss, Kay Savik, David M Radosevich. The incontinence-associated dermatitis and its severity instrument: development and validation. J Wound Ostomy Continence Nurs. Sep-Oct 2010;37(5):527-35.

Karen Van den Bussche, Sofie Verhaeghe , Ann Van Hecke, Dimitri Beeckman. The Ghent Global IAD Monitoring Tool (GLOBIAD-M) to monitor the healing of incontinence-associated dermatitis (IAD): Design and reliability study. Int Wound J. 2018 Aug;15(4):555-564. doi: 10.1111/iwj.12898. Epub 2018 May 24.
