# Supplementary material for: Benefits of a digital health technology for older nursing home residents. A de-novo cost-effectiveness model for digital health technologies to aid in the assessment of toileting and containment care needs
Source: PLoS One. 2024 Jan 2;19(1):e0295846. doi: 10.1371/journal.pone.0295846 (PMC10760782; doi:10.1371/journal.pone.0295846)
Supplement: S3 File — (PDF) [file pone.0295846.s004.pdf]

Estimates for time spent on continence care were derived from a report by Knibbe and Knibbe (2020). They report on secondary analyses of existing Dutch datasets on continence and related subjects, which were used in previous studies with a maximum age of five years. The datasets contained data on a total of 5,122 incontinent clients, distributed over home care (n=1,412), care homes (n=1,766) and nursing homes (n=1,944). Patients under the age of 65 years were excluded, as were patients in acute care, general hospitals, psychiatric care and care for people with disabilities.

Table 1. Time demand for carers of continence care episodes

N = 4102<br>Minutes per 24 hours per client	Time required/ per episode (total care time) for toileting	Time required/ per episode (total care time) for toileting plus pad change	Time required/ per episode (total care time) limited to pad change (bed or toilet)	Time required for checking of saturation or presence of faeces	Time needed for solving leakage problems (changing bedlinen and/or clothes)
Care dependent living at home	7,3 minutes	9,3 minutes	4,2	Not by professional.	4,3
Care dependent living in an institution; care homes	9,5	11,5	6,6	3,2	7,4
Care dependent living in an institution; nursing home	11,7	18,2	10,2	4,1	9,1

NB: total care time is defined as the sum of the total time carers need for this procedure. If 2 carers are involved the sum of their total time was used to calculate the average care time.
