# Supplementary material for: Benefits of a digital health technology for older nursing home residents. A de-novo cost-effectiveness model for digital health technologies to aid in the assessment of toileting and containment care needs
Source: PLoS One. 2024 Jan 2;19(1):e0295846. doi: 10.1371/journal.pone.0295846 (PMC10760782; doi:10.1371/journal.pone.0295846)
Supplement: S4 File — (PDF) [file pone.0295846.s005.pdf]

Table S1. Care Stage thresholds - Approach

Resident profile	Average product use over 24 hrs	Rothwell Value per Product	24-hour total absorbency range (Rothwell value per Product * # units)
Care Stage 1			
Larger residents	<2 products of ‘Plus’ (= ‘Ultra’) absorbency, L-XL	2250	<4500
Average	<2 products of ‘Plus’ (= ‘Ultra’) absorbency, M-R	1900	<3800
Care Stage 2			
Larger residents	2 - 3 products of ‘Plus’ (= ‘Ultra’) absorbency, L-XL	2250	4500 - 6750
Average	2 - 3 products of ‘Plus’ (= ‘Ultra’) absorbency, M-R	1900	3800 - 5700
Care Stage 3			
Larger residents	> 3 products of ‘Plus’ (= ‘Ultra’) absorbency, L-XL	2250	> 6750
Average	> 3 products of ‘Plus’ (= ‘Ultra’) absorbency, M-R	1900	> 5700

Keys: L-XL = Large to Extra-Large size; M-R = Medium to Regular size

Notes: Typical absorbency usage is given as equivalent units of ‘Plus’ / ‘Ultra’ absorbency: this is the most common absorbency level used for CS2, although those in CS1 may use lower absorbency products and those in CS3 may user higher.

Thresholds are calculated according to expected usage for residents who are either light user (Care Stage 1: less than two ‘Plus’ [2250 RV for Large size] products, or equivalent in total absorbency, on average over 24 hours); those with typical requirements (Care Stage 2: two to three ‘Plus’ products over 24 hours); and higher users (Care Stage 3: more than three products over 24 hours)

The Rothwell value of the respective absorbent product is a laboratory measure described in the ISO 11948-1 standard and measures the maximum theoretical absorption capacity of the absorbing material in the entire absorbent product. Measuring the actual product weight is the preferred measure of incontinence severity, but in everyday practice this method is labour intensive and not widely used.

Since the primary source of effectiveness evidence in this health economic analysis was based on the ARCTICC study, using the ISO 11948-1 method was the best available proxy to measure incontinence severity.

Table S2 Group transition probabilities

Transition pr. G1		
From	To	Pr.
**G1_CS1**	G2_CS1	0.003101
	G2_CS2	0.027907
	G3_CS1	0.002204
	G3_CS2	0.019833
	G1_CS1	0.946955
**G1_CS2**	G2_CS2	0.027907
	G2_CS3	0.003101
	G3_CS2	0.019833
	G3_CS3	0.002204
	G1_CS2	0.946955
**G1_CS3**	G2_CS3	0.031008
	G3_CS3	0.022037
	G1_CS3	0.946955
**Transition pr. G2**		
From	To	Pr.
**G2_CS1**	G3_CS1	0.00633
	G3_CS2	0.056971
	G4_CS1	0.000178
	G4_CS2	0.001603
	G2_CS1	0.934918
**G2_CS2**	G3_CS2	0.056971
	G3_CS3	0.00633
	G4_CS2	0.001603
	G4_CS3	0.000178
	G2_CS2	0.934918
**G2_CS3**	G3_CS3	0.063301
	G4_CS3	0.001781
	G2_CS3	0.934918
**Transition pr. G3**		
From	To	Pr.
**G3_CS1**	G4_CS1	0.006446
	G4_CS2	0.058016
	G5_CS1	0.002204
	G5_CS2	0.019833
	G3_CS1	0.913501
**G3_CS2**	G4_CS2	0.058016
	G4_CS3	0.006446
	G5_CS2	0.019833
	G5_CS3	0.002204
	G3_CS2	0.913501
**G3_CS3**	G4_CS3	0.064462
	G5_CS3	0.022037
	G3_CS3	0.913501

**Transition pr. G4**		
From	To	Pr.
**G4_CS1**	G5_CS1	0.058493
	G5_CS2	0.526433
	G6_CS1	0.002204
	G6_CS2	0.019833
	G4_CS1	0.393038
**G4_CS2**	G5_CS2	0.526433
	G5_CS3	0.058493
	G6_CS2	0.019833
	G6_CS3	0.002204
	G4_CS2	0.393038
**G4_CS3**	G5_CS3	0.584925
	G6_CS3	0.022037
	G4_CS3	0.393038
**Transition pr. G5**		
From	To	Pr.
**G5_CS1**	G6_CS1	0.002204
	G6_CS2	0.019833
	G5_CS1	0.977963
**G5_CS2**	G6_CS2	0.019833
	G6_CS3	0.002204
	G5_CS2	0.977963
**G5_CS3**	G6_CS3	0.022037
	G5_CS3	0.977963
**Transition pr. G6**		
From	To	Pr.
**G6_CS1**	G6_CS1	1
**G6_CS2**	G6_CS2	1
**G6_CS3**	G6_CS3	1

Table S3 Canadian unit costs

Input parameter	Unit cost, in CAD 2020	Source
**Staff costs, per minute**	$ 0.34	[51]
**Acquisition costs DHT (software + 1 assessment per resident/year), per day**	$ 0.70	Company
**Products conventional care CS1, per cycle**	$ 1.17	ARCTICC study
**Products conventional care CS2, per cycle**	$ 2.07	
**Products conventional care CS3, per cycle**	$ 2.78	
**Products DHT CS1, per cycle**	$ 1.29	
**Products DHT CS2, per cycle**	$ 1.84	
**Products DHT CS3, per cycle**	$ 2.54	
**Perineal hygiene conventional care, per application**	$ 0.07	[22]
**Perineal hygiene DHT, per application**	$ 0.18	[22]
**Skin treatment SHL2-4, per application**	$ -	[41]
**Skin treatment SHL5, per application**	$ 0.34	[53]
**Treatment PU1, per event**	$ 2,167	[56]
**Treatment PU2, per event**	$ 8,966	
**Treatment PU3, per event**	$ 14,896	
**Treatment PU4, per event**	$ 21,491	
**Treatment constipation, per event**	$ 10.65	[55]
**Treatment UTI, per event**	$ 4.12	[31]
**Treatment fracture, per event**	$ 6,215.35	[54]
**Disposal cost per kg incontinence-associated waste**	$ 0.23	[66] [52]

Table S4 Pressure Ulcer Grade 1 to 4

Grade 1	Non-blanchable erythema of intact skin. Discolouration of the skin, warmth, oedema, induration or hardness may also be used as indicators, particularly on individuals with darker skin.
Grade 2	Partial thickness skin loss involving epidermis, dermis or both. The ulcer is superficial and presents clinically as an abrasion or blister.
Grade 3	Full thickness skin loss involving damage to or necrosis of subcutaneous tissue that may extend down to, but not through underlying fascia.
Grade 4	Extensive destruction, tissue necrosis, or damage to muscle, bone, or supporting structures with or without full thickness skin loss.

Source: European Pressure Ulcer Advisory Panel (EPUAP) (www.epuap.com)

Table S5 Reduction (%) in care consequences with DHT

Group	Estimate[1]	2	3	4	5	6
**Estimated improvement per group[2]**		21.7%	21.7%	21.7%	21.7%	21.7%
**Fractures**	7.5%	1.6%	1.6%	1.6%	1.6%	1.6%
**UTI**	37.5%	8.2%	8.2%	8.2%	8.2%	8.2%
**Constipation**	75.0%	16.3%	16.3%	16.3%	16.3%	16.3%
**PU**	5.00%	1.1%	1.1%	1.1%	1.1%	1.1%
Sources: Expert opinion (N=1), 2022; Swedish pilot study						

[1]Estimated reduction per care consequence was based on the median of effect ranges based on expert opinion (N=1).

[2]The effect of the intervention on care consequence was based on the percentage increase in toileting visits derived from a pilot study on the intervention in Sweden and was assumed across groups. Since the estimated reductions per care consequence were the maximum possible reductions if an individual would be 100% continent, the effect size from the DHT on toileting visits was multiplied by the estimate to arrive at the relative reduction in care consequences due to the DHT.

Table S6 Frequencies DHT

		Group 2			Group 3			Group 4			Group 5			Group 6		
Frequencies per 24H	**Source**	**CS 1**	**CS 2**	**CS 3**	**CS 1**	**CS 2**	**CS 3**	**CS 1**	**CS 2**	**CS 3**	**CS 1**	**CS 2**	**CS 3**	**CS 1**	**CS 2**	**CS 3**
**Products used**	ARCTICC study	1.52	2.51	3.53	1.52	2.51	3.53	1.52	2.51	3.53	1.52	2.51	3.53	1.52	2.51	3.53
**Product weight**	ARCTICC study	146.41	239.46	345.99	146.41	239.46	345.99	146.41	239.46	345.99	146.41	239.46	345.99	146.41	239.46	345.99
**Perineal hygiene (prevention)**	Assumption: same as Products used 24H	1.52	2.51	3.53	1.52	2.51	3.53	1.52	2.51	3.53	1.52	2.51	3.53	1.52	2.51	3.53
**Product checks**	ARCTICC study	0.86	1.06	1.58	0.86	1.06	1.58	0.86	1.06	1.58	0.86	1.06	1.58	0.86	1.06	1.58
**Product change (incl. perineal hygiene)**	ARCTICC study	1.52	2.51	3.53	1.52	2.51	3.53	1.52	2.51	3.53	1.52	2.51	3.53	1.52	2.51	3.53
**Toileting assistance**	[24] Expert opinion (G6) Estimated CS2-3	2.26	1.37	0.97	2.26	1.37	0.97	2.26	1.37	0.97	2.26	1.37	0.97	0.00	0.00	0.00
**Product leakage requiring clothes change and/or bedlinen change**	ARCTICC study	0.09	0.32	0.39	0.09	0.32	0.39	0.09	0.32	0.39	0.09	0.32	0.39	0.09	0.32	0.39
**Treatment application SHL2-4**	[41]	0.00	0.00	0.00	0.00	0.00	0.00	0.00	0.00	0.00	0.00	0.00	0.00	0.00	0.00	0.00
**Treatment application SHL5**	[41]	1.52	2.51	3.53	1.52	2.51	3.53	1.52	2.51	3.53	1.52	2.51	3.53	1.52	2.51	3.53

Table 1 Frequencies conventional care

		Group 2			Group 3			Group 4			Group 5			Group 6		
	**Source**	**CS 1**	**CS 2**	**CS 3**	**CS 1**	**CS 2**	**CS 3**	**CS 1**	**CS 2**	**CS 3**	**CS 1**	**CS 2**	**CS 3**	**CS 1**	**CS 2**	**CS 3**
**Products used**	ARCTICC study	1.50	2.51	3.53	1.50	2.51	3.53	1.50	2.51	3.53	1.50	2.51	3.53	1.50	2.51	3.53
**Product weight**	ARCTICC study	140.49	247.12	362.18	140.49	247.12	362.18	140.49	247.12	362.18	140.49	247.12	362.18	140.49	247.12	362.18
**Perineal hygiene (prevention)**	Assumption: same as Products change 24H	1.50	2.51	3.53	1.50	2.51	3.53	1.50	2.51	3.53	1.50	2.51	3.53	1.50	2.51	3.53
**Checks**	ARCTICC study	1.14	1.01	1.65	1.14	1.01	1.65	1.14	1.01	1.65	1.14	1.01	1.65	1.14	1.01	1.65
**Product change (incl. perineal hygiene)**	ARCTICC study	1.50	2.51	3.53	1.50	2.51	3.53	1.50	2.51	3.53	1.50	2.51	3.53	1.50	2.51	3.53
**Toileting assistance**	[24] Expert opinion (G6) Estimated CS2-3	1.90	1.13	0.81	1.90	1.13	0.81	1.90	1.13	0.81	1.90	1.13	0.81	0.00	0.00	0.00
**Product leakage requiring clothes change**	ARCTICC study	0.49	0.59	0.35	0.49	0.59	0.35	0.49	0.59	0.35	0.49	0.59	0.35	0.49	0.59	0.35
**Treatment application SHL2-4**	[41]	0.00	0.00	0.00	0.00	0.00	0.00	0.00	0.00	0.00	0.00	0.00	0.00	0.00	0.00	0.00
**Treatment application SHL5**	[41]	1.50	2.51	3.53	1.50	2.51	3.53	1.50	2.51	3.53	1.50	2.51	3.53	1.50	2.51	3.53
